# 

**Published:** 1881-07

**Authors:** 


### AMERICAN DENTAL ASSOCIATION.

The next annual meeting of the American Dental Association will be held in Irving Hall, corner of Irving Place and Fifteenth Street, New York City, commencing at 10 o'clock A. M., on Tuesday, July 12, 1881.

GEO. H. CUSHING, Rec. Sec'y.

After the expediency of the change of time of the meeting of the Association, as above announced, was suggested, consultation was had with as many of the members of the Association, by letter and otherwise, as the limited time would permit; and, from information thus obtained, it was believed that the best interests of the Association would be subserved, and the wishes of a large majority carried out, by antedating the time of meeting to the 12th of July.

The principal reason for this change lies in the fact that a large number of the permanent working members have already made arrangements to attend the Medical Congress to be held in London at the usual time of the meeting of the Association, many of whom, we are informed, would attend our meeting at this earlier date. Believing that the number of members in attendance would be very much larger by this arrangement, and the meeting more generally satisfactory, the officers and executive committee, after much deliberation, have assumed the responsibility of calling the Association together as announced above.

C. N. PIERCE, President.
GEO. H. CUSHING, Secretary.
W. H. GODDARD, Treasurer.
J. N. CROUSE, Chairman.
W. H. MORGAN,
C. N. PIERCE,
C. S. STOCKTON,
GEO. W. KEELY,
S. G. PERRY,
CHAS. D. COOK,
G. V. BLACK,
Executive Committee.

The local Committee of Arrangements would recommend the Westminster Hotel, corner of Sixteenth Street and Irving Place, as a very quiet and good hotel, and as being in close proximity to the place of meeting. They have reduced the price from their regular rates of $3.50 to $2.50 per day.

If any members desire to secure rooms, and will write, the committee will secure them.

A. L. NORTHROP, 44 W. Forty-Sixth St., N. Y.

W. H. ATKINSON, 41 E. Ninth St., N. Y.

GEO. A. MILLS, 169½ Columbia Heights, Brooklyn, N. Y.

Committee of Arrangements.

### NATIONAL DENTAL ASSOCIATION.

The regular annual meeting of the Dental Association of the United States of America will be held in New York city, commencing Monday, August 8th, 1881.

This Association was organized, not in opposition to those already existing, but to supply a want that is not met by any one of them. It is so constituted that members of the profession in all parts of the country can in turn attend its meetings and participate in its control and its benefits.

All members of the profession are therefore cordially invited to attend this meeting. R. FINDLEY HUNT, Sec'y,
Washington, D. C.

### CONTENTS.

### COMMUNICATIONS.

A MONTHLY MAGAZINE DEVOTED TO THE INTERESTS OF

**THE DENTAL PROFESSION.**

*TERMS REDUCED TO $2.50 PER YEAR. SINGLE COPIES 25C.*

EDITED BY PROF. J. TAFT, D.D.S.

AND

*Published by SPENCER, CROCKER & CO., Ohio Dental Depot.*

The volume commences in January and closes in December.

The REGISTER being issued monthly is always fresh, therefore Indispensable to the practitioner who desires to keep posted up with the new inventions and improvements which are daily developed. Neither expense nor effort will be spared to give value and variety to its contents. Articles will be illustrated with well executed engravings whenever they will add to the interest or assist to explanation.

Its extensive circulation and frequency of issue make it one of the BEST DENTAL ADVERTISING MEDIUMS in the United States.

All subscriptions must begin with January or July.

Local or special notices, five lines or less, will be inserted for$3 each insertion; over five lines, 50 cts. per line.

All advertising bills payable in advance, or at furthest, after the issue of the first number containing the advertisement. All transient advertisements to be paid for in advance.

☞CONTRIBUTIONS SOLICITED.

Exclusively Editorial Communications should be addressed to Editor.

The latest number of the REGISTER may be ordered with or without other goods at any time, and all letters should be addressed to

***SPENCER, CROCKER & CO., Ohio Dental Depot, Cincinnati, O.***

